# Brain white matter plasticity and functional reorganization underlying the central pathogenesis of trigeminal neuralgia

**DOI:** 10.1038/srep36030

**Published:** 2016-10-25

**Authors:** Tian Tian, Linying Guo, Jing Xu, Shun Zhang, Jingjing Shi, Chengxia Liu, Yuanyuan Qin, Wenzhen Zhu

**Affiliations:** 1 Department of Radiology, Tongji Hospital, Tongji Medical College, Huazhong University of Science and Technology, Wuhan 430030, People’s Republic of China; 2 Department of Neurology, Tongji Hospital, Tongji Medical College, Huazhong University of Science and Technology, Wuhan 430030, People’s Republic of China

## Abstract

Peripheral nerve damage does not fully explain the pathogenesis of trigeminal neuralgia (TN). Central nervous system changes can follow trigeminal nerve dysfunction. We hypothesized that brain white matter and functional connectivity changes in TN patients were involved in pain perception, modulation, the cognitive-affective system, and motor function; moreover, changes in functional reorganization were correlated with white matter alterations. Twenty left TN patients and twenty-two healthy controls were studied. Diffusion kurtosis imaging was analyzed to extract diffusion and kurtosis parameters, and functional connectivity density (FCD) mapping was used to explore the functional reorganization in the brain. In the patient group, we found lower axial kurtosis and higher axial diffusivity in tracts participated in sensory, cognitive-affective, and modulatory aspects of pain, such as the corticospinal tract, superior longitudinal fasciculus, anterior thalamic radiation, inferior longitudinal fasciculus, inferior fronto-occipital fasciculus, cingulated gyrus, forceps major and uncinate fasciculus. Patients exhibited complex FCD reorganization of hippocampus, striatum, thalamus, precentral gyrus, precuneus, prefrontal cortex and inferior parietal lobule in multiple modulatory networks that played crucial roles in pain perception, modulation, cognitive-affective system, and motor function. Further, the correlated structural-functional changes may be responsible for the persistence of long-term recurrent pain and sensory-related dysfunction in TN.

Trigeminal neuralgia (TN) is a unilateral prevalent pain disorder characterized by brief electric shock-like pains, sudden in onset and termination and limited to the allocation of one or more divisions of the trigeminal nerve. According to the new International Headache Society classification of TN, the pain is usually evoked by trivial stimuli such as shaving, washing, talking, smoking and/or brushing the teeth (trigger factors) and continually occurs spontaneously^1^. The pains usually moderate for variable periods. Between paroxysms, patients are usually asymptomatic.

Arterial compression of the trigeminal nerve at the root entry zone is the widely accepted cause of TN^2^, having received significant support from researchers, especially surgical and radiological scientists^3^. The root entry zone corresponds to the transition zone between central and peripheral myelin where the trigeminal nerve is believed to be the most vulnerable to focal myelin loss or damage caused by extrinsic compression^4^. However, numerous studies have already shown that TN pathophysiology cannot be entirely explained by a nerve-vessel-conflict^5^,^6^. Theories concerning central nervous system (CNS) pathogenesis of TN have been proposed, accounting for both the abnormal generation of sensory impulses and their spread from fibers to pathways involved in the perception and modulation of pain in the brain^7^.

Many neuroimaging studies have also used highly sensitive MRI methods to evaluate the structural or functional brain changes in TN patients. Diffusion tensor imaging (DTI) has been shown to be a useful tool for identifying microstructural abnormalities in TN, including the root entry zone and brain white matter abnormalities^8^,^9^,^10^,^11^,^12^. Previous studies have addressed morphological changes occurring in not only grey matter volume^13^ but also in the cortical thickness of central structures^14^,^15^. These abnormal brain regions in TN patients have been associated with pain perception, modulation, the cognitive-affective system, and motor function, further reflecting the maladaptive brain plasticity due to long-term nociceptive inputs. In a study that combined cortical thickness and brush-induced allodynic activation, the authors reported that changes in the cortical thickness of TN patients were frequently correlated with functional allodynic activations^16^. Based on described studies, both structural plasticity and functional reorganization may explain the CNS pathogenesis that contributes to the unique TN symptoms.

As an extension of DTI, diffusion kurtosis imaging (DKI) has been proposed to characterize non-Gaussian diffusion by estimating the excess kurtosis of the displacement distribution^17^,^18^. Based on DKI data, both diffusion and kurtosis parameters, which are especially suitable for evaluating microstructural integrity in white matter regions with complex fiber arrangement, could be obtained^19^. DKI has exhibited improved sensitivity and specificity in detecting developmental and pathological changes in neural tissues relative to conventional DTI^20^,^21^,^22^,^23^,^24^. In addition, functional connectivity density (FCD) mapping is a newly developed data-driven method to identify the distribution of functional connectivity regions in the human brain. This ultrafast technique allows the calculation of functional connectivity maps in brain networks, overcoming the limitations of seed-based approaches for functional study^25^. In the present study, we examined both DKI and FCD in TN using multiple MRI technologies to provide insight into its central mechanisms. We hypothesized that brain white matter and functional connectivity changes in TN patients were involved in pain perception, modulation, the cognitive-affective system, and motor function; moreover, changes in functional reorganization were correlated with white matter alterations. The correlated brain structural-functional changes may be responsible for the persistence of long-term recurrent pain and sensory-related dysfunction in TN patients.

## Results

### Diffusion parameters from DKI

**Figure 1:**
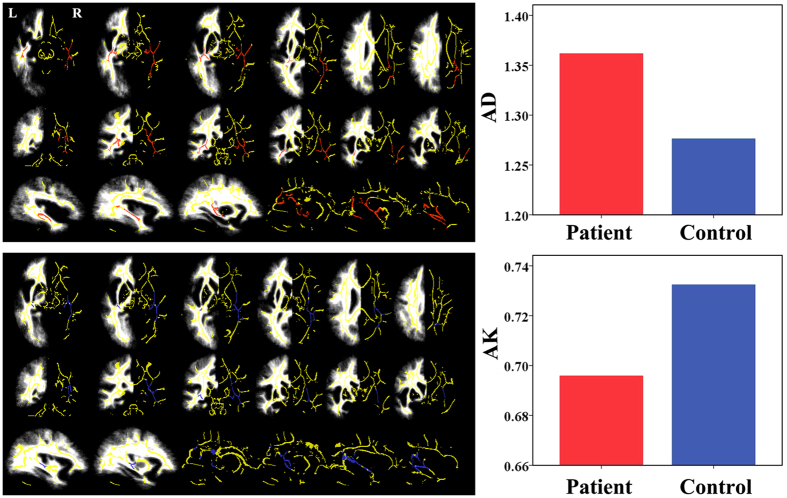
The white-matter fibers with significant intergroup differences (*P * < 0.05, FWE corrected) in DKI-derived diffusion and kurtosis parameters. Compared with healthy controls, TN patients exhibited increased AD and decreased AK in white matter regions involved in the sensory, cognitive-affective, and modulatory aspects of pain. Bars represent the means. AD values are given in 10^−3^ square millimeters per second (μm^2^/ms). AD = axial diffusivity, AK = axial kurtosis, DKI = diffusion kurtosis imaging, FWE = family-wise error, L = left, R = right, TN = trigeminal neuralgia.

The white matter fibers with significant intergroup differences (*P* < 0.05, FWE corrected) in DKI-derived diffusion parameters are shown in Fig. 1. Compared with healthy controls, TN patients exhibited increased axial diffusivity (AD) relative to healthy controls in the right cingulated gyrus, bilateral superior longitudinal fasciculus, bilateral anterior thalamic radiation, forceps major, bilateral inferior longitudinal fasciculus, bilateral inferior fronto-occipital fasciculus, and bilateral uncinate fasciculus. However, there were no significant differences in fractional anisotropy (FA), radial diffusivity (RD), and mean diffusivity (MD) between the two groups.

### Kurtosis parameters from DKI

The white matter fibers with significant intergroup differences (*P* < 0.05, FWE corrected) in DKI-derived kurtosis parameters are shown in Fig. 1. Compared with healthy controls, TN patients exhibited significantly decreased axial kurtosis (AK) in white matter regions with fiber arrangement, such as in the right corticospinal tract, right superior longitudinal fasciculus, bilateral anterior thalamic radiation, bilateral inferior longitudinal fasciculus, and bilateral inferior fronto-occipital fasciculus. However, there were no significant differences in radial kurtosis (RK) and mean kurtosis (MK) between the two groups.

### Functional changes from FCD mapping

**Figure 2:**
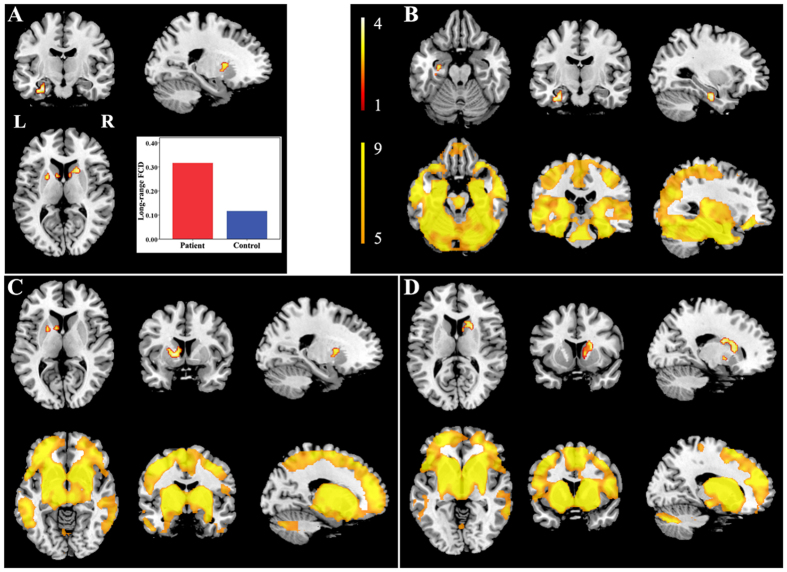
Brain regions with increased long-range FCDs in TN patients. Compared with healthy controls, TN patients exhibited significantly increased long-range FCDs in the left hippocampus (**B**) and bilateral striatum (**C,D**). The rsFC pattern for each significant cluster is shown below the cluster. FCD = functional connectivity density, L = left, R = right, rsFC = resting state functional connectivity, TN = trigeminal neuralgia.

**Figure 3:**
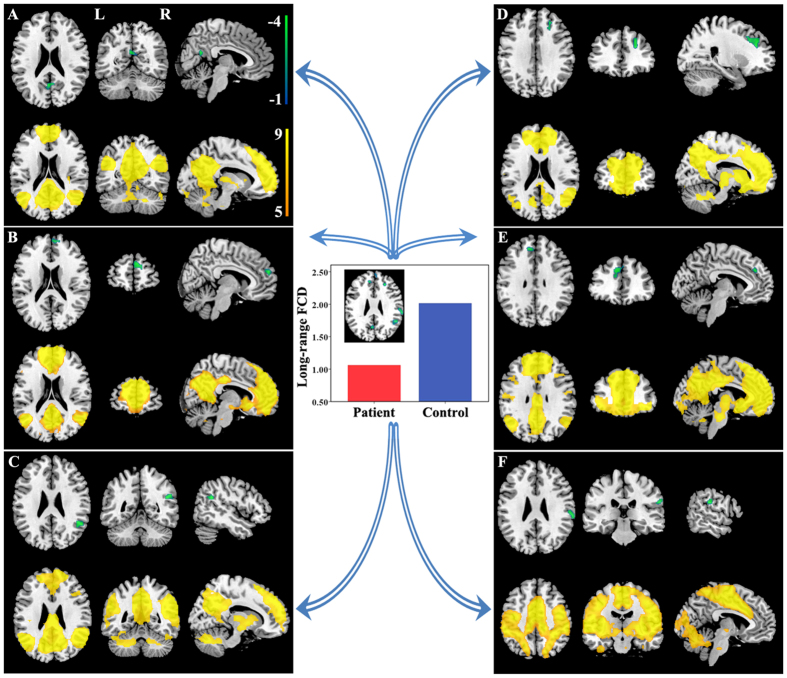
Brain regions with decreased long-range FCDs in TN patients. Compared with healthy controls, TN patients exhibited significantly decreased long-range FCDs in the bilateral precuneus (**A**), bilateral PFC (**B,D,E**), right angular gyrus (**C**), and right supramarginal gyrus (**F**). The rsFC pattern for each significant cluster is shown below the cluster. FCD = functional connectivity density, L = left, PFC = prefrontal cortex, R = right, rsFC = resting state functional connectivity, TN = trigeminal neuralgia.

Brain regions with significant long-range FCD differences are shown in Figs 2 and 3. Compared with healthy controls, TN patients exhibited significantly increased long-range FCDs in the left hippocampus (Fig. 2B) and bilateral striatum (Fig. 2C,D). TN patients also exhibited significantly decreased long-range FCDs in the bilateral precuneus (Fig. 3A), bilateral prefrontal cortex (PFC) (Fig. 3B,D,E), right angular gyrus (Fig. 3C), and right supramarginal gyrus (Fig. 3F). The resting state functional connectivity (rsFC) pattern of each significant cluster is shown under the cluster. We found that the left hippocampus was a component of the reward-emotion network; that the caudate and putamen were parts of the striatal network; that the precuneus, bilateral PFC, and right angular gyrus were parts of the default mode network (DMN); and that the right supramarginal gyrus was part of the sensorimotor network (SMN).

**Figure 4:**
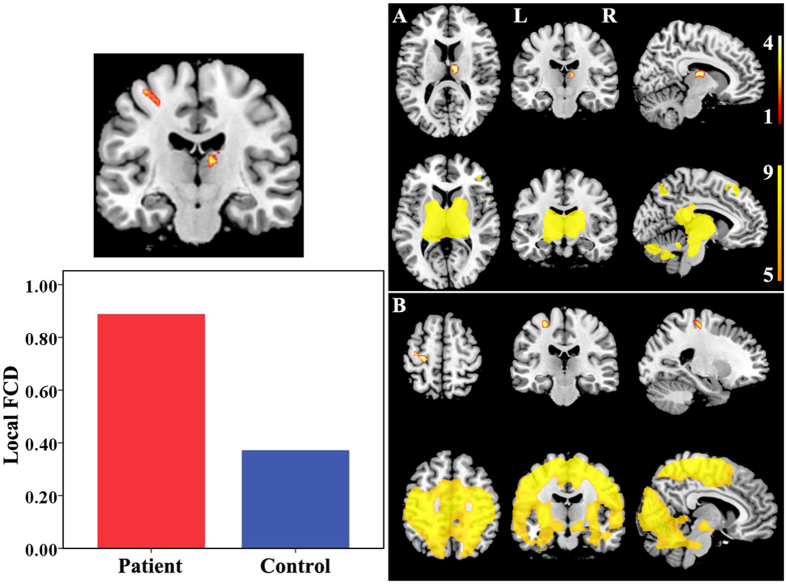
Brain regions with increased local FCDs in TN patients. Compared with healthy controls, TN patients exhibited significantly increased local FCDs in the right thalamus (**A**) and left precentral gyrus (**B**). The rsFC pattern for each significant cluster is shown below the cluster. FCD = functional connectivity density, L = left, R = right, rsFC = resting state functional connectivity, TN = trigeminal neuralgia.

**Figure 5:**
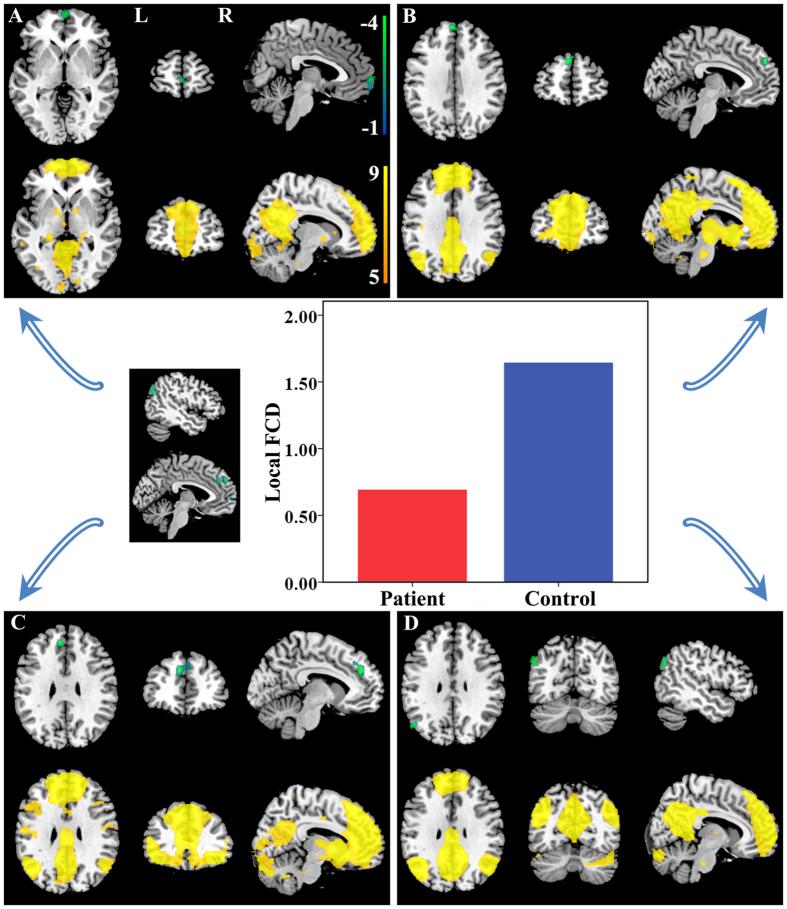
Brain regions with decreased local FCDs in TN patients. Compared with healthy controls, TN patients exhibited significantly decreased local FCDs in the bilateral medial PFC (**A–C**) and left angular gyrus (**D**). The rsFC pattern for each significant cluster is shown below the cluster. FCD = functional connectivity density, L = left, PFC = prefrontal cortex, R = right, rsFC = resting state functional connectivity, TN = trigeminal neuralgia.

Brain regions with significant local FCD differences are shown in Figs 4 and 5. Compared with healthy controls, TN patients exhibited significantly increased local FCDs in the right thalamus (Fig. 4A) and left precentral gyrus (Fig. 4B). TN patients also exhibited significantly decreased local FCDs in the bilateral medial PFC (Fig. 5A–C) and left angular gyrus (Fig. 5D). The rsFC pattern of each significant cluster is shown under the cluster. We found that the right thalamus was part of the thalamus network; that the left precentral gyrus was part of the SMN; and that the medial PFC and left angular gyrus were components of the DMN.

### Correlations between functional and structural changes in TN patients

**Figure 6:**
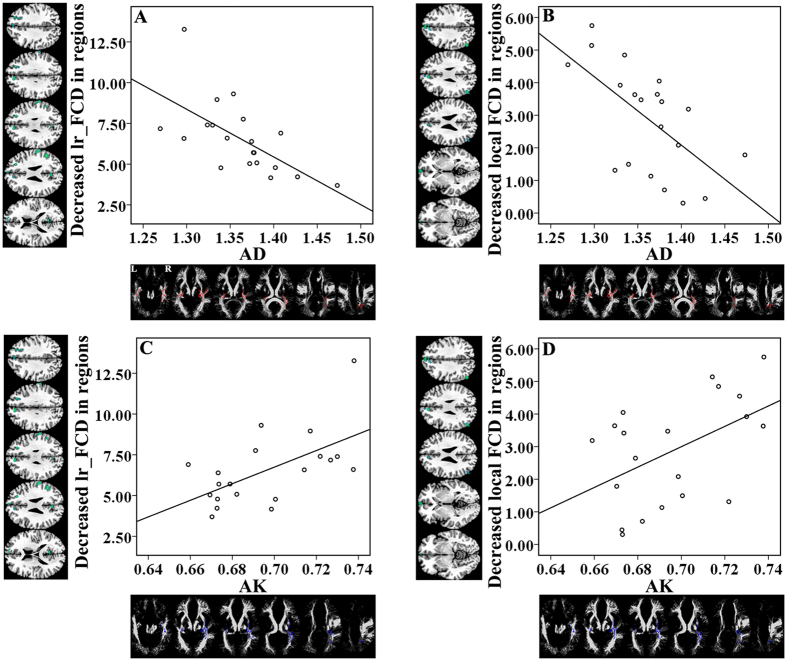
Correlations between functional and structural changes in TN patients. The total decreased long-range FCD of the bilateral precuneus, bilateral PFC, right angular gyrus, and right supramarginal gyrus were significantly correlated with AD (r = −0.632, *P* = 0.003) and AK (r = 0.586, *P* = 0.007) changes, respectively, in the white matter regions of TN patients. The total decreased local FCD of the left angular gyrus and bilateral PFC were significantly correlated with AD (r = −0.614, *P* = 0.004) and AK (r = 0.488, *P* = 0.029) changes, respectively, in the white matter regions of TN patients. AD = axial diffusivity, AK = axial kurtosis, L = left, FCD = functional connectivity density, lr_FCD = long-range functional connectivity density, PFC = prefrontal cortex, R = right, TN = trigeminal neuralgia.

To test correlations between functional and structural changes, we calculated the AD, AK, and FCD of clusters that showed significant intergroup differences and investigated correlations between functional (FCD) and structural (AD and AK) changes using the general linear model. The total decreased long-range FCDs of the bilateral precuneus, bilateral PFC, right angular gyrus, and right supramarginal gyrus were significantly correlated with AD (r = −0.632, *P* = 0.003) and AK (r = 0.586, *P* = 0.007) changes, respectively, in the white matter of TN patients. The total decreased local FCDs of the left angular gyrus and PFC were significantly correlated with AD (r = −0.614, *P* = 0.004) and AK (r = 0.488, *P* = 0.029) changes, respectively, in the white matter of TN patients. Further details are shown in Fig. 6. Increased long-range and local FCDs in regions showed no significant correlation with AD and AK changes in the white matter of TN patients. No significant correlations were obtained between functional and structural changes in healthy controls.

### Associations between the imaging measures and clinical data

A general linear model was applied to explore the correlations between imaging measures (FCD, AD, and AK) and clinical data in TN patients. TN patients were divided into two subgroups based on visual analog scale (VAS) scores. For moderate pain patients (VAS scores of 4–7), increased local FCDs in the right thalamus and left precentral gyrus were positively correlated with total descriptors (r = 0.905, *P* = 0.002) (Figure S1), and AK changes in the white matter were negatively correlated with VAS scores (r = −0.923, *P* = 0.001) (Figure S2). For extreme patients (VAS scores of 8–10), no significant correlations were found between clinical data and brain changes.

## Discussion

In this study, we performed a comprehensive analysis combining DKI and resting-state functional magnetic resonance imaging (fMRI) data to investigate imaging determinants of the pathogenic mechanisms of TN in the CNS. We found that TN patients showed both white matter plasticity and functional reorganization in the sensory, cognitive-affective, and modulatory aspects of pain. Moreover, AD and AK changes were significantly correlated with FCD changes. These results provided evidence of the structural-functional changes underlying CNS pathogenesis in TN patients.

Although TN is known to involve trigeminal nerve dysfunction, brain white matter plasticity can occur following peripheral nerve damage^7^. In this study, we reported white matter abnormalities in brain regions of TN patients, mainly marked by lower AK in the right corticospinal tract, right superior longitudinal fasciculus, bilateral anterior thalamic radiation, bilateral inferior longitudinal fasciculus, and bilateral inferior fronto-occipital fasciculus. We also found higher AD in the right cingulated gyrus, bilateral superior longitudinal fasciculus, bilateral anterior thalamic radiation, forceps major, bilateral inferior longitudinal fasciculus, bilateral inferior fronto-occipital fasciculus, and bilateral uncinate fasciculus. We attributed the increased AD and decreased AK to water molecule diffusion strengthening and regrouping in the axial direction. The corpus callosum interconnects the cerebral hemispheres, and the splenium allows for the rapid communication and integration of sensory information^26^. The superior longitudinal fasciculus, thought to play an important role in higher-order cognitive function, has shown abnormal pain processing in TN patients^8^. The inferior fronto-occipital fasciculus links cortical regions involved in the processing of pain, emotion, and rewards^27^. Diffusion alterations in the uncinate fasciculus have been correlated with pain experience and perception^28^. Microstructural alterations of the anterior thalamic radiation and cingulated gyrus connect cortical regions also involved in body perception. The inferior longitudinal fasciculus has been correlated with pain severity^29^. The corticospinal tract is known to play a role in pain control^30^. In chronic pain disorder, the imbalance of descending pain modulation in the corticospinal tract has been linked to higher trait anxiety^31^. Together, these tracts connect brain regions known to participate in sensory, cognitive affective, and modulatory aspects of pain. Abnormal increased diffusion in these tracts may result in alterations in information integration. Finally, the prolonged nociceptive information to the CNS could lead to central sensitization^32^, which is a process involving central neuroinflammation induced by chronic pain attacks^33^. This inflammation may, in turn, contribute to the unique symptoms of TN pain.

The hippocampus has been positively connected to regions linked to reward and emotion, including the limbic system, amygdala, midbrain, pons, striatum, thalamus, and insula^34^. The role of the hippocampus in pain modulation has been demonstrated during pain inhibition^35^ and learning mechanisms involved in the emergence of persistent pain^36^. It was reported that the decreased functional connectivity between the hippocampus and the rostral anterior cingulate cortex represented an attenuated defensive response to nociceptive pain^37^. Functional connectivity in the striatum has been identified during sensory-motor, cognitive and limbic loops. Both the caudate nucleuses and hippocampus are involved in cognitive learning as well as pain modulation^38^. The putamen and thalamus were reported to play a pivotal role in the genesis and maintenance of TN pain^39^. Furthermore, it is worth mentioning the sensory gating function of the thalamus that controls the flow of sensorimotor information to and from the cerebral cortex^40^. Our study provided evidence for higher FCDs of the hippocampus, thalamus, and striatum in TN patients. It is possible that the long-lasting TN pain created more demands for pain modulation, expressed as relatively increased FCDs in these affective-cognitive and sensory modulation pathways. The DMN has been known to be altered in pain diseases and to be involved in cognitive and memory-related aspects of pain modulation^41^,^42^,^43^. It was reported that the neuropathic pain engaged brain regions critical for cognitive assessment, emphasizing the unique role of the PFC in pain states^44^. The medial PFC activation in the expectation of pain intensity indicated complex modulation to adjust sensory, cognitive and motor systems for adequate neural and behavioral responses^45^. As another critical part in the DMN, the precuneus has shown decreased connectivity with several regions of pain processing, reward, and higher executive functioning within the prefrontal and parietal cortices^46^. The angular gyrus, moreover, is a main part of the inferior parietal lobule (IPL), which is involved in several cognitive domains and important nodes of the DMN. The IPL has consistently been found to be involved in attentive control and the response to salient events^47^. Shifting or distracting attention away from pain is a well-known central analgesia mechanism involving the descending pain modulatory system^45^ and may elicit an analgesic effect linked to the memory processing function of DMN. In our study, decreased FCDs in the DMN regions suggestive of sparse brain connectivity were observed in patients. The impaired connectivity in those regions may affect pain regulation by the DMN. On the other hand, we found functional modulatory disequilibrium in regions involved in the SMN. The highly connected precentral gyrus involved in motor function may indicate an enhanced motor output in TN patients. The decreased FCDs in the pain perception of the supramarginal gyrus may not only influence large-scale rapid communication with other regions but also facilitate the generation or maintenance of hyperalgesic states^48^. Together, our findings show that multiple modulatory networks play crucial roles in the adaptive and maladaptive modulations of the pain experience. Complex functional reorganization of these areas involved in pain perception, modulation, cognitive-affective system, and motor function may reflect vulnerability to TN development. The precise network changes in the CNS pathogenesis of TN remains to be further investigated.

Our findings indicate that the abnormal increased diffusion in white matter tracts involved in sensory, cognitive-affective, and modulatory aspects of pain are correlated with decreased FCDs in pain perception and modulation in TN patients. These correlated changes may be responsible for the persistence of long-term recurrent pain and sensory-related dysfunction in TN patients. Interestingly, we found that the degree of pain was positively correlated with increased diffusion of white matter and increased local FCDs of the thalamus and precentral gyrus, providing imaging parameters that may be used as useful clinical biomarkers in patients with moderate pain.

This study has several limitations that should be addressed in future work. First, to our knowledge, many brain regions critical to functional changes in pain modulation, such as the anterior cingulate cortex and brainstem, which have been extensively studied, were not found to be involved in pain modulation in this study. The precise functional changes of TN remain to be further investigated. Second, changes in white matter observed in our study were not fully consistent with results from previous DTI research that showed widespread regions of FA, RD, and MD abnormalities in TN patients^8^. It should be noted that the FA and related indices that are obtained with DKI are substantially different from that are obtained with DTI^17^. As an extension of DTI, DKI has been proposed to characterize non-Gaussian diffusion by estimating the excess kurtosis of the displacement distribution^17^,^18^. The acquisition of diffusion-weighted images with two or more nonzero b values along 15 or more diffusion-encoding directions (for each b value) is required for DKI. Based on DKI data, both diffusion and kurtosis parameters, which are especially suitable for evaluating microstructural integrity in white matter regions with complex fiber arrangement, could be obtained^19^. DKI has exhibited improved sensitivity and specificity in detecting developmental and pathological changes in neural tissues relative to conventional DTI^20^,^21^,^22^,^23^,^24^. Thus, these discrepancies with previous DTI research may due to the differences in b values, diffusion-encoding directions, processing methods, and various pathological processes of samples. Finally, the number of subjects in our sample was small. A large sample of TN patients should be recruited to verify our DKI findings in future research.

In summary, this comprehensive study focused on the imaging determinants of the pathogenic mechanisms of TN in the CNS. We found increased diffusion in white matter fibers involved in the sensory, cognitive-affective, and modulatory aspects of pain, which transform nociceptive information to the CNS. The functional reorganization of brain regions involved in multiple modulatory networks of pain perception, modulation, the cognitive-affective system, and motor function was complex. Decreased FCDs were significantly correlated with increased diffusion in white matter fibers. The degree of pain was positively correlated with white matter and FCD changes in moderate pain patients. These correlated changes may be responsible for the persistence of long-term recurrent pain and sensory-related dysfunction in TN patients. These findings may contribute to the development of CNS treatment options in neuropathic pain.

## Methods

### Subjects

The human experiment was approved by the Ethical Committee of Tongji Hospital of Tongji Medical College of Huazhong University of Science and Technology, and written informed consent was obtained from each subject before the study. All methods were carried out in accordance with approved institutional guidelines and regulations. A total of 20 right-handed patients (12 females and 8 males; mean age: 52.6 years; range: 36 to 65 years) were included in this study. Inclusion criteria were left paroxysmal attacks of pain lasting from a fraction of a second to 2 minutes, affecting one or more divisions of the trigeminal nerve; intense, sharp, superficial or stabbing paroxysmal pain precipitated from trigger areas or by trigger factors; stereotyped attacks in the individual patient; no clinically evident neurological deficits; pain not attributed to another disorder; and no previous surgical procedures. Every patient was asked to complete the short form of the McGill Pain Questionnaire. This questionnaire consisted of 15 descriptors (11 sensory and 4 affective) rated along an intensity scale, VAS, and present pain intensity (PPI) scale. Further details are shown in the Supplementary Information. Twenty-two matched healthy subjects (16 females and 6 males; mean age: 52.2 years; range: 38 to 63 years) were also recruited as controls. The demographic and clinical data for our sample are provided in Table 1.

### Image acquisition

MRI was performed using a 3.0-Tesla MR system (Discovery MR750, General Electric, Milwaukee, WI, USA). Tight but comfortable foam padding was used to minimize head motion, and earplugs were used to reduce scanner noise. DKI data were acquired by a spin-echo single-shot echo planar imaging sequence with the following parameters: repetition time = 6500 ms; echo time = 85 ms; matrix = 128 × 128; field of view = 256 × 256 mm; in-plane resolution = 2 × 2 mm; slice thickness = 3 mm without gap; 46 axial slices; 25 encoding diffusion directions with two values of b (b = 1250 and 2500 s/mm^2^) for each direction and 2 non-diffusion weighted images (b = 0 s/mm^2^). All images were visually inspected to ensure that only images without visible artifacts were included in subsequent analyses. Resting-state fMRI data were obtained using the single-shot echo planar imaging sequence with the following imaging parameters: repetition time/echo time = 2000/35 ms; field of view = 240 mm × 240 mm; matrix = 64 × 64; flip angle = 90°, slice thickness = 4 mm; no gap; 40 transversal slices; 240 volumes.

### Calculation of diffusion and kurtosis parameters

DKI is an extension of DTI. It provides a more accurate model of diffusion for quantifying the deviation from a Gaussian distribution, which is known as kurtosis. By acquiring data for at least two nonzero diffusion gradient factors (b value) in more than 15 nonlinear directions, the kurtosis metrics and conventional diffusion metrics (including MK, AK, RK, MD, AD, RD and FA) are obtained. AK is parallel to the main direction of diffusion, RK is perpendicular to the main direction of diffusion, and MK is the average kurtosis of all diffusion directions. AK might reflect the axonal integrity and density of fiber bundles, RK might reflect the myelin integrity and axonal density, and MK might reflect microstructural complexity.

Eddy current-induced distortion and motion artifacts in the DKI dataset were corrected using affine alignment of each diffusion weighted image to the b = 0 image. After skull-stripping, a diffusion kurtosis estimator was implemented to calculate the diffusion and kurtosis tensors using the constrained linear least squares-quadratic programming algorithm^49^.

### Tract-Based Spatial Statistics (TBSS)

The following steps were adopted for the TBSS analysis^50^. All subjects’ FA images were aligned to a template of averaged FA images (FMRIB-58) in the Montreal Neurological Institute (MNI) space using a non-linear registration algorithm. After transformation into the MNI space, a mean DKI_FA image was created and thinned to generate a mean DKI_FA skeleton of the white matter tracts. Each subject’s DKI_FA image was then projected onto the skeleton via filling the mean DKI_FA skeleton with DKI_FA values from the nearest relevant tract center by searching perpendicular to the local skeleton structure for the maximum DKI_FA value. The registration and projection data derived from the DKI_FA analysis were then applied to the other parametric images of each subject to ensure an exact spatial correspondence of the different parameters.

Voxel-wise statistical analysis across subjects on the skeleton space was carried out using a permutation-based inference tool for nonparametric statistics. Group comparisons between TN patients and healthy controls were performed using a general linear model with age and gender as covariates of no interest. The mean DKI_FA skeleton was used as a mask, and the number of permutations was set to 5000. To simultaneously control for both type I and type II errors, the significance threshold was determined with a *P* < 0.05 (two-tailed) after correcting for family-wise error (FWE) using the threshold-free cluster enhancement (TFCE) option.

### Functional data preprocessing, FCD calculation and statistical analysis

The first 10 volumes for each subject were discarded. Head motion parameters were estimated, and each volume was realigned to the mean whole volume map to correct for geometrical displacements using a six-parameter rigid-body transformation. Data were excluded from further analysis if their maximum displacement in any of the orthogonal directions (x, y, z) was greater than 2 mm or if there was a maximum rotation (x, y, z) greater than 2.0°. We also calculated framewise displacement (FD)^51^, which indexes volume-to-volume changes in head position. There was no significant group difference in FD. Individual functional images were spatially normalized to the MNI space. The functional images were then re-sampled into a voxel size of 3 × 3 × 3 mm^3^. Finally, the datasets were bandpass filtered with frequencies ranging from 0.01 to 0.08 Hz, and several nuisance covariates (six motion parameters and average blood oxygen level-dependent (BOLD) signals of the ventricular and white matter) were regressed out from the data.

We calculated the FCD of each voxel using the in-house script that was written in the Linux platform according to the method described by Tomasi and Volkow^25^. Pearson’s linear correlation was used to calculate the functional connections, and two voxels with a correlation coefficient R > 0.6 were considered functionally connected. The calculation of the FCD was restricted to voxels in the grey matter regions having a signal-to-noise ratio >50% to minimize unwanted effects from susceptibility-related signal-loss artifacts. The local FCD at a given voxel x0 was computed as the local k(x0) between x0 and its neighboring voxels using a “growing” algorithm developed in the Linux platform. Specifically, a voxel (xj) was added to the list of voxels functionally connected with x0 only if it was adjacent to a voxel that was linked to x0 by a continuous path of functionally connected voxels with R_0j_ > 0.6. This calculation was repeated for all voxels that were adjacent to voxels that belonged to the list of voxels functionally connected to x0 in an iterative manner until no new voxels could be added to the list. The local FCD at x0 was computed as the number of elements in the local functional connectivity cluster, k(x0). Next, the calculation was initiated for a different x0 and then finally applied to all qualified voxels of the brain. The global FCD at a given voxel x0 was computed as the global number of functional connections, k(x0), between x0 and all other voxels. This calculation was repeated for all x0 voxels in the brain. The strength of the long-range FCD was equated to [global FCD–local FCD] to remove all connected voxels belonging to the local cluster. To increase the normality of the distribution, grand mean scaling of local and long-range FCDs was performed by dividing by the mean value of the qualified voxels of the whole brain. Finally, the normalized FCDs were smoothed using a Gaussian kernel of 6 × 6 × 6 mm^3^ full-width at half-maximum.

We compared FCD differences between TN patients and healthy controls in a voxel-wise manner using two-sample t-tests. To control for age- and sex-related changes, age and sex were implemented in our statistical model as covariates. The FWE small-volume correction (*P* < 0.05) was used to correct for multiple comparisons. To determine the functional networks to which brain regions with significant differences in FCDs belonged, we defined these regions as regions of interest (ROIs) for the whole-brain rsFC analysis. The ROI-based whole-brain rsFC analyses were performed in the Supplementary Information.

### Correlation analysis

The structural and functional changes in each cluster that showed significant group differences were extracted. To further analyze the correlations between structural/functional changes and clinical data, including disease duration and the short form of the McGill Pain Questionnaire, we applied a general linear model to investigate correlations between brain changes (changes in FCD, DKI parameters) and clinical data. To explore the extent to which FCD differences were driven by the corresponding structural differences, we also used this model to test correlations between these functional and structural changes in TN patients.

## Additional Information

**How to cite this article**: Tian, T. *et al*. Brain white matter plasticity and functional reorganization underlying the central pathogenesis of trigeminal neuralgia. *Sci. Rep.* **6**, 36030; doi: 10.1038/srep36030 (2016).

## Supplementary Material

Supplementary Information

## Figures and Tables

**Table 1:** Demographic and clinical data for TN patients and healthy controls.

	TN	Healthy controls
Number of patients	20	22
Gender (female/male)	12/8	16/6
Age (years)	52.6 ± 8.9 (range: 36–65)	52.2 ± 6.1 (range: 38–63)
Duration (months)	21.1 ± 16.2 (range: 2–60)	NA
VAS	7.7 ± 1.6	NA
PPI	3.3 ± 1.1	NA
PRI (Affective)	3.1 ± 1.9	NA
PRI (Sensory)	10.7 ± 5.0	NA
PRI (Total)	14.7 ± 4.9	NA

NA = not applicable, PPI = present pain intensity, TN = trigeminal neuralgia, VAS = visual analogue scale/score, PRI = pain rating index.
