# Supplementary material for: Brain white matter plasticity and functional reorganization underlying the central pathogenesis of trigeminal neuralgia
Source: Sci Rep. 2016 Oct 25;6:36030. doi: 10.1038/srep36030 (PMC5078771; doi:10.1038/srep36030)
Supplement: Supplementary Information [file srep36030-s1.doc]

**Supplementary Information**

**Title：Brain white matter plasticity and functional reorganization underlying the central pathogenesis of trigeminal neuralgia**

Tian Tian^1^, Linying Guo^1^, Jing Xu^2^, Shun Zhang^1^, Jingjing Shi^1^, Chengxia Liu^1^, Yuanyuan Qin^1^, Wenzhen Zhu^1^*

1 Department of Radiology, Tongji Hospital, Tongji Medical College, Huazhong University of Science and Technology, Wuhan 430030, People’s Republic of China

2 Department of Neurology, Tongji Hospital, Tongji Medical College, Huazhong University of Science and Technology, Wuhan 430030, People’s Republic of China

Tian Tian and Linying Guo contributed equally to the work.

Correspondence to Wenzhen Zhu, MD,

Department of Radiology, Tongji Hospital, Tongji Medical College, Huazhong University of Science and Technology

1095 Jiefang Ave, Wuhan, Hubei, People’s Republic of China, 430030

E-mail: zhuwenzhen@hotmail.com

**Supplementary Methods**

**The short form of the McGill Pain Questionnaire**

Every patient was asked to complete the short form of the McGill Pain Questionnaire. This questionnaire consisted of 15 descriptors (11 sensory and 4 affective) rated along an intensity scale, visual analog scale (VAS), and present pain intensity (PPI) scale. VAS for pain intensity was a simple straight line approximately 10-cm long extending from “no pain” to the extreme limit of pain. Patients were asked to score the pain according to their own perception. The PPI is a six-point verbal rating scale. In this scale, patients are asked to choose the word that best describes the overall intensity of their pain ranging from “none” (0 score) to the worst “excruciating” (5 score). Further details were provided in Table S1.

**The regions of interest (ROI)-based whole-brain resting state functional connectivity (rsFC) analyses**

The ROI-based whole-brain rsFC analyses were performed as follows: (1) for each subject, the correlation coefficient between the mean time series of each ROI and that of each voxel in the whole brain was computed and converted into a z value to improve normality using the Fisher’s r-to-z transformation; and (2) individuals’ z values were entered into a random-effect one-sample t-test to identify the brain regions that were significantly correlated with the ROI. The significant rsFC maps were corrected for multiple comparisons using the FWE (P ＜ 0.05) method. On the basis of the rsFC patterns of these ROIs, we may infer the possible network to which each ROI belonged.


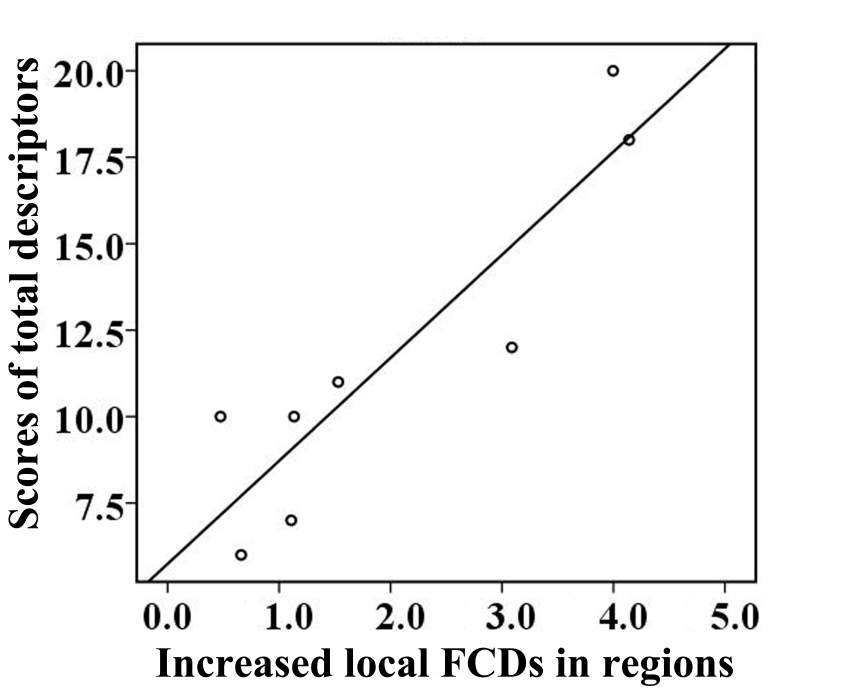


**Figure S1** For moderate pain patients (VAS scores of 4-7), increased local FCDs in the right thalamus and left precentral gyrus were positively correlated with scores of total descriptors. FCD = functional connectivity density; VAS = visual analogue scale/score.


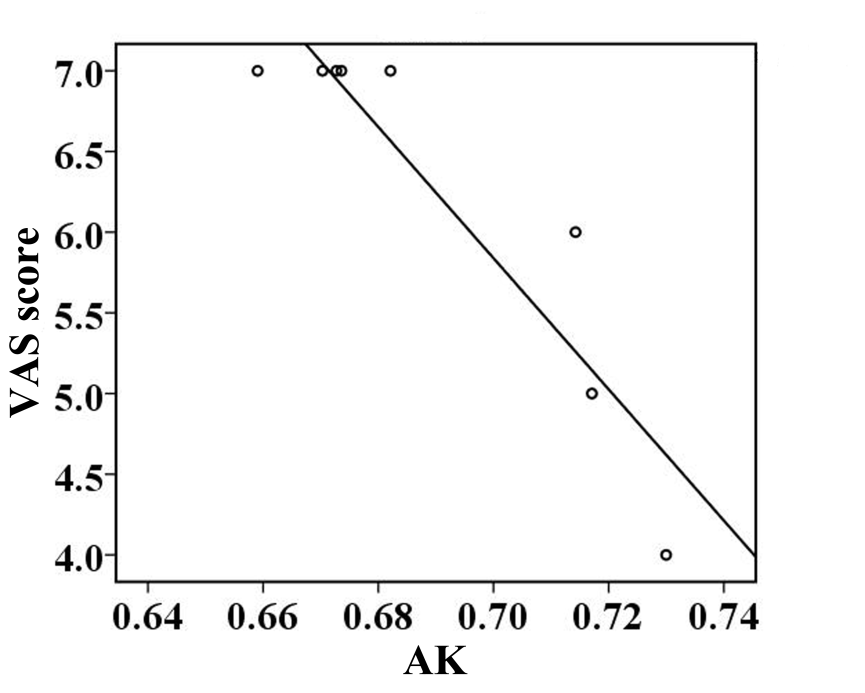


**Figure S2** For moderate pain patients (VAS scores of 4-7), AK changes in the white matters were negatively correlated with VAS scores. AK = axial kurtosis; VAS = visual analogue scale/score.

**Table S1** The Short-form of McGill Pain Questionnaire

Patient’s Name:_______________________ Date:______________________

|  | None | Mild | Moderate | Severe |
| --- | --- | --- | --- | --- |
| Throbbing | 0) | 1) | 2) | 3) |
| Shooting | 0) | 1) | 2) | 3) |
| Stabbing | 0) | 1) | 2) | 3) |
| Sharp | 0) | 1) | 2) | 3) |
| Cramping | 0) | 1) | 2) | 3) |
| Gnawing | 0) | 1) | 2) | 3) |
| Hot – burning | 0) | 1) | 2) | 3) |
| Aching | 0) | 1) | 2) | 3) |
| Heavy | 0) | 1) | 2) | 3) |
| Tender | 0) | 1) | 2) | 3) |
| Splitting | 0) | 1) | 2) | 3) |
| Tiring – exhausting | 0) | 1) | 2) | 3) |
| Sickening | 0) | 1) | 2) | 3) |
| Fearful | 0) | 1) | 2) | 3) |
| Punishing-Cruel | 0) | 1) | 2) | 3) |

*Visual Analogue Scale (VAS)*

Using a vertical line, please mark your current level of pain on the line below.

No pain |_________________________________________________| Extreme pain

PPI

0 No pain — — — —

1 Mild — — — —

2 Discomforting — — — —

3 Distressing — — — —

4 Horrible — — — —

5 Excruciating — — — —
